# On the Illumination of the Cavities of the Body by Means of Electricity

**Published:** 1861-02

**Authors:** M. Fossagrives


On the Illumination of the Cavities of the Body by Means of Electricity.—By *M. Fossagrives.*—A long time ago, the author had conceived the idea that the electrical light might be advantageously substituted in diagnostic researches, or in operative manœuvres, for the ordinary methods of illumination, which are either insufficient in intensity, or defective by the color of the light, or embarrassing by the impossibility of using them without interfering with the space required for instruments, and by the necessity, on account of the heat evolved, of keeping the light at a great distance from the surface to be illuminated. The whole problem consisted in discovering a source of light, with little or no caloric action, which might be condensed in tubes of small size and of diversified form, and which would be of sufficient whiteness not to alter materially the color of the organic textures lighted up by means of it. By the assistance of M. Th. du Moncel and M. Ruhmkorff, this problem seems to have been solved in a satisfactory manner. M. du Moncel, having observed that the vacuum tubes of Geissler do not become heated under the influence of the electric light transmitted through them, and knowing, moreover, that this light is more brilliant in proportion as the tubes of communication between the terminal balls of the apparatus are of a smaller diameter, suggested that, in taking an apparatus of that kind, in which a long tube, almost capillary in size, should be bent upon itself, and convoluted in the manner of the electro-magnetic multipliers, we might obtain not only a kind of luminous cylinder, capable of being introduced into narrow cavities, but even a kind of electrical beacon on certain points of which the light might be concentrated, without any risk either of over-heating or of commotions of any kind. The first part of the problem was, therefore, solved. With regard to the color of the light in the tubes, as this depends entirely on the nature of the gas on which the vacuum has been made, and as the color is white, with certain mixed gases, as carburretted hydrogen, carbonic acid, hydrochloric acid, etc., all that is required to meet this part of the problem is to prepare the tubes with suitable gases. M. Ruhmkorff, to whom the construction of these tubes was entrusted, and who has introduced several improvements in their formation, has obtained results which are quite satisfactory. He has found out a mixture of gas, which gives a suitable white light in the tubes; and experience has shown that the amount of light afforded by the apparatus is more than sufficient for the requirements of medicine and surgery.

Without for the present tracing absolutely the field of application of this new means of illumination, the following may nevertheless be pointed out:—1. As a means of diagnostic exploration, in the examination of accessible organic passages, for the purpose of recognizing their normal or pathological condition. 2. As a means of illumination to assist experimental action. It is easy to foresee the utility of this means in those operations which present, among their greatest difficulties, the impossibility of lightening up suitably the surfaces on which instruments are to act. In particular, the following will derive advantage from this new application: 1st, Staphyloraphy; 2d, Operations for vesico-vaginal fistula; 3d, Extirpation of naso-pharyngeal or uterine polypi. 4th, Excision of the tonsils, etc. Certain dental operations, also, may be expected to be rendered more easy of execution by this proceeding. It may be questioned, also, whether the field of the retina might not be illuminated more easily and completely by the same means.—*Acad. des Sciences*, Jan., 1860.

